# PMINR: Pointwise Mutual Information-Based Network Regression – With Application to Studies of Lung Cancer and Alzheimer’s Disease

**DOI:** 10.3389/fgene.2020.556259

**Published:** 2020-10-15

**Authors:** Weiqiang Lin, Jiadong Ji, Yuchen Zhu, Mingzhuo Li, Jinghua Zhao, Fuzhong Xue, Zhongshang Yuan

**Affiliations:** ^1^Department of Biostatistics, School of Public Health, Cheeloo College of Medicine, Shandong University, Jinan, China; ^2^Department of Data Science, School of Statistics, Shandong University of Finance and Economics, Jinan, China; ^3^Cardiovasucular Epidemiology Unit, Department of Public Health and Primary Care, University of Cambridge, Cambridge, United Kingdom

**Keywords:** biological networks, pointwise mutual information, regression, lung cancer, Alzheimer’s disease

## Abstract

Complex diseases are believed to be the consequence of intracellular network(s) involving a range of factors. An improved understanding of a disease-predisposing biological network could lead to better identification of genes and pathways that confer disease risk and therefore inform drug development. The group difference in biological networks, as is often characterized by graphs of nodes and edges, is attributable to effects of these nodes and edges. Here we introduced pointwise mutual information (PMI) as a measure of the connection between a pair of nodes with either a linear relationship or nonlinear dependence. We then proposed a PMI-based network regression (PMINR) model to differentiate patterns of network changes (in node or edge) linking a disease outcome. Through simulation studies with various sample sizes and inter-node correlation structures, we showed that PMINR can accurately identify these changes with higher power than current methods and be robust to the network topology. Finally, we illustrated, with publicly available data on lung cancer and gene methylation data on aging and Alzheimer’s disease, an evaluation of the practical performance of PMINR. We concluded that PMI is able to capture the generic inter-node correlation pattern in biological networks, and PMINR is a powerful and efficient approach for biological network analysis.

## Introduction

A complex disease is understood to be the consequence not of abnormality involving a single biomolecule (e.g., RNA, protein, metabolite) but of their network(s) and possibly a variety of other factors (Barabási et al., 2011). Biomolecules interact with each other in such network(s) which underpin the disease pathogenesis and progression. Specific types of networks (e.g., protein–protein interaction networks) are often used to represent a given type of biological processes, each containing information about levels and inter-relationships among specific biomolecules (Albert, 2005). A recent gene set analysis method has also emphasized the importance of incorporating network or pathway information (Li et al., 2019). Indeed, it is uncommon to observe that a significant gene-disease association disappears when studied within a network or pathway, and vice versa. Consequently, there is a framework of “think globally, act locally” in great need to develop statistical methods to detect whether specific biological network is strongly associated with the disease outcome. It is thus more appropriate to investigate how the biological networks vary with disease status, rather than analyze factors individually. A greater understanding of the role of biological network(s) in disease etiology and treatment should lead to better identification of disease-related genes and pathways, and consequently to more precise targets for drug development.

A biological network is commonly described as a graph such that nodes (or vertices) are used to represent biomolecules and edges to represent consequences or physiological interactions between vertices. In general, both the node effects (e.g., the magnitude of each gene’s expression in regulation network) and the edge effects (e.g., the strength of connection) can contribute to the disease. A given biological network is characterized with respect to what the nodes represent and what the nature of the interactions is between these nodes (edges) (Sonawane et al., 2019). For instance, a protein–protein interaction network describes proteins as well as physiological interactions between them, while a gene co-expression network involves genes and their expression patterns. For the latter, the impact of a specific genetic abnormality is unrestricted to the activity of a single gene in question but able to spread along its connections with other genes and propagate through interactions to involve other genes in the network. The graph abstraction has greatly facilitated the study of networks.

It is particularly challenging to quantify inter-node connection strength precisely with a unified metric, especially when involving group (e.g., patients versus healthy controls) differences in biological networks (Gambardella et al., 2013; Yates and Mukhopadhyay, 2013; Ruan et al., 2015). In an attempt to accommodate changes in nodes and edges which lead to network differences, we previously developed statistics to test the group difference for weighted biological networks (Ji et al., 2016), for pathways with chain structure (Ji et al., 2015; Yuan et al., 2016a) and for directed biological networks (Yuan et al., 2016b). Nevertheless, these methods have little capacity to adjust for potential confounding factors and covariates (e.g., age, sex, batch effect), which served as a motivation for the current investigation into network regression techniques to infer the effect of a biological network as a whole (i.e., treating the whole network as the independent variables), accounting for the potential confounders through a regression model. As will soon become clear, this is furnished via two steps, the first of which is to find an appropriate metric to measure the inter-node connection that can better reflect the underlying relationships among the network nodes, to be followed at the second step by a unified regression framework involving both the nodes and the edges; together they bring dependence structures into inference and achieve high statistical efficiency.

In more detail, our approach is concerned about regression methodology for assessing relationships between disease outcome and a particular biological network with adjustment for potential confounding factors. Below we first introduce pointwise mutual information (PMI) to measure the strength of connection between a pair of nodes in the network, as currently PMI is commonly used in machine learning and text mining (Turney, 2001; Read, 2004) to capture linear or nonlinear relationships between two nodes. We then construct the PMI-based network regression (PMINR) model for a given network to identify differential patterns of network changes (with respect to both nodes and edges) responsible for complex traits or disease. Extensive simulations were conducted to evaluate the performance of our model, including the robustness and power of PMINR. Finally, publicly available data on lung cancer and gene methylation data on aging and Alzheimer’s disease from the Religious Orders Study and Memory and Aging Project (ROSMAP) study were analyzed to evaluate the practical performance of PMINR. Our focus here on logistic regression for its broad applicability in biomedical research can be easily extended to generalized linear models involving a variety of outcomes.

## Materials and Methods

The PMI of two node variables *X* and *Y* can be defined as follows (Church and Hanks, 1990):

(1) $$P\,M\,I\,\left(x,y\right)=\log\frac{p\,\left(x,y\right)}{p\,\left(x\right)\,p\,\left(y\right)}$$

where *p*(*x*,*y*)is the joint distribution of *X* and *Y*, *p*(*x*) and *p*(*y*)their marginal distributions. Statistically, the stronger the correlation between two nodes regardless of linear or nonlinear relationship, the more deviation PMI from 0, when if and only if *X* and *Y* are independent. Thus, PMI, to some extent, is a non-independence metric. To make the estimator of the joint density of two nodes variable more robust, we choose bivariate kernel density estimation (BKDE) for PMI. Let *X*, *Y* be a bivariate sample drawn from a common distribution described by the density function*f*. The BKDE is defined as

(2) $${\hat{f}}_{\text{H}}\,\left(z;\text{H}\right)=\frac{1}{n}\,\sum_{i=1}^{n}K_{H}\,\left(\text{z}-{\text{Z}}_{i}\right)$$

where **z** = (*x*,*y*)^*T*^ and **Z**_*i*_ = (*X*_*i*_,*Y*_*i*_)^*T*^, *i* = 1, 2,…,*n*, and **H** is the bandwidth (or smoothing) 2×2 matrix which is symmetric and positive definite; *K* is the bivariate kernel function which is a symmetric multivariate density and *K*_**H**_(**z**) = |**H**|^−1/2^*K*(**H**^−1/2^**z**). For the present study, we use the bivariate normal kernel:

(3) $$K_{H}\,\left(z\right)={\left(2\,\pi \right)}^{-d/2}\,{\left|H\right|}^{-1/2}\,\exp\left(-\frac{1}{2}\,z^{T}\,{\text{H}}^{-1}\,z\right)$$

Assume that we have a biological network with *p* nodes measured over individuals. For individual *l*(*l* = 1,2,…,*N*), Let

$$Y_{l}=\left\{ \begin{array}{cc} 0\; & l\in g\,r\,o\,u\,p\,0\\ 1\; & l\in g\,r\,o\,u\,p\,1 \end{array}\right.$$

be the binary response variable, *Z*_*s*_(*s* = 1,…,*S*) be the covariates (e.g., age, gender). The PMINR is defined as:

(4) $$\text{logit}\left(P\left(Y=1\right)\right)=\beta_{0}+\sum_{s=1}^{s}\alpha_{s}Z_{s}+\sum_{i=1}^{p}\beta_{i}x_{i}+\sum_{i=1}^{p}\sum_{j>i}^{p}I_{i\,j}\gamma_{i\,j}E_{i\,j}$$

where *x*_*i*_ denotes the *i*th node,

$$I_{i\,j}=\left\{ \begin{array}{cc} 0\; & x_{i}\,\text{and}\,x_{j}\,\mathrm{are}\,\mathrm{unconnected}\,\mathrm{in}\,\mathrm{the}\,\mathrm{network}\\ 1\; & \text{otherwise} \end{array}\right.$$

is an indicator variable, *E*_*ij*_ denotes the estimator of PMI between node *x*_*i*_ and node *x*_*j*_ using BKDE, respectively. The regression coefficients are denoted by α_*s*_, β_*i*_ and γ_*ij*_. Here, we use the cubic- and quadratic-spline interpolation to construct the BKDE-based estimator of PMI. PMINR naturally decomposed the change of the whole network into the node changes and edge changes. Using a likelihood ratio test, it can test whether the whole network is significantly associated with the response variable, and using a Wald test it can detect identify which nodes or edges are related to the response variable.

### Simulation

**FIGURE 1:**
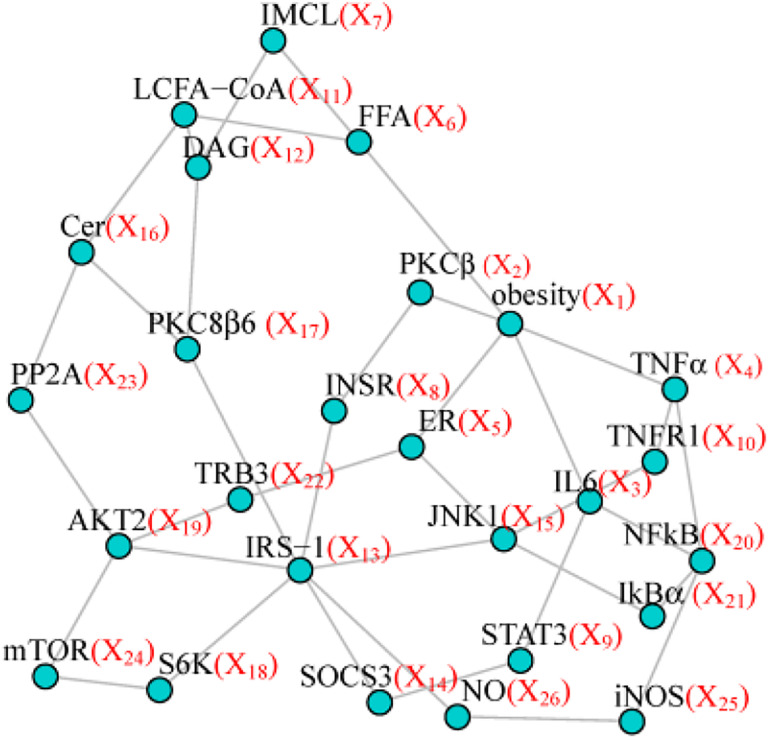
The simulated network structure based on the Insulin resistance pathway from KEGG.

To make our simulation more realistic, we set as our model network the topological structure from the pathway of insulin resistance downloaded from Kyoto Encyclopedia of Genes and Genomes (KEGG) including 26 nodes and 37 edges (Figure 1). Four simulation scenarios under different sample sizes and variable inter-node correlation patterns (see the details below), were designed to assess the type I error and statistical power. Specifically, we used Wald test to assess the type I error for testing one randomly selected node without any effect (node test), or one randomly selected edge without any effect (edge test). We used Wald test to assess the power for testing the effecting node, or the effecting edge, or the effecting pairs of node and edge. We compared PMINR with three other methods, including

• the product moment network regression (PMNR) which uses the common linear correlation to represent the between-node connection strength.
• the DGCA method which is differential gene correlation analysis (i.e., edge effect) to assess the difference in gene-gene regulatory relationships under different conditions (McKenzie et al., 2016).
• the RANK method which can detect the whole pathway due to either correlations or mean changes (Alvo et al., 2010).

Each scenario included four situations: (1) only nodes of network having the effect, (2) only edges of network having effects, (3) both nodes and edges having effects, with the nodes not hanging on the edge (e.g., node X_6_ and edge E_4,10_ in Figure 1), (4) both the nodes and edges having the effects, with the nodes hanging on the edge (e.g., X_4_ and E_4,10_ in Figure 1).

In scenario 1, we generated data using the linear correlation to represent the network edge and evaluate the performance of all these four methods. We randomly assigned the effecting node and edge for the four aforementioned situations, respectively. The simulated *m*-dimensional node variables were generated from a multivariate normal distribution *N*_*m*_(0,Σ) with covariance matrix Σ using the R package *mvtnorm*. We specified the covariance matrix Σ = (*I*_*ij*_ρ_*ij*_)_*m*×*m*_, where

$$I_{i\,j}=\left\{ \begin{array}{cc} 1, & E_{i\,j}\in E\,\left(G\right)\\ 0, & E_{i\,j}\notin E\,\left(G\right) \end{array}\right.$$

*i*≠*j*,*i*,*j* = 1,2,…,*m* is the indicator function, *m* = 26, ρ_*ij*_ is assigned by randomly choosing a number from 0.1 to 0.55 with a step 0.05 and the eigenvalues are calculated to judge whether the covariance matrix is positive definite. We generated the response variable *Y* from binomial distribution with

$$P\left(Y=1\right)=\frac{\exp\left(\beta_{0}+\sum_{i=1}^{m}\beta_{i}\,X_{i}+\sum_{i=1}^{m}\sum_{j>i}^{m}I_{i\,j}\,\gamma_{i\,j}\,E_{i\,j}\right)}{1+\exp\left(\beta_{0}+\sum_{i=1}^{m}\beta_{i}\,X_{i}+\sum_{i=1}^{m}\sum_{j>i}^{m}I_{i\,j}\,\gamma_{i\,j}\,E_{i\,j}\right)}$$

where *X*_*i*_ and *E*_*ij*_ denotes the different vertices and edges between two groups (case *vs.* control), β_*i*_andγ_*ij*_ denote the corresponding effect size on *Y*. We set the intercept to be zero to make the two groups (case vs control) have equal sample size when the global network has no effect on the response variable. The type I error rate was assessed by setting all node and edge parameters to be 0, β_*i*_ = 0, γ_*ij*_ = 0, *i*,*j* = 1,2,…,*m*. We further assessed the power by setting β = 0.3, γ = 0.2. Here, we randomly selected an effecting node or an effecting edge, or an effecting pair of node and edge in each replication to minimize the impact of network structure, randomly selecting the effecting nodes and edges can avoid subjectiveness of the design and make the results more convincing.

We further considered three other patterns of nonlinear relationships between the network nodes, $X_{j}=X_{i}^{2}$ (scenario 2), *X*_*j*_ = *sin*⁡*X*_*i*_ (scenario 3), *X*_*j*_ = (*sin*⁡*X*_*i*_)^2^ (scenario 4). The data were generated based on the pre-defined nonlinear relationship. For instance, if we assign the sine relationship between node *X*_4_ and node *X*_10_, then *X*_10_ = α*sin⁡*X*_4_ + ε, the parameter α was used to represent the nonlinear connection strength between *X*_4_ and *X*_10_. Note that the nonlinear sine relationship between *X*_*4*_and*X*_*10*_can be depicted by the linear relationship between *sin*⁡*X*_4_ and *X*_*10*_. We set *E*_4,10_ = α*sin⁡*X*_4_*X_10_ to generate the response *Y*. All regression coefficients were set to be 0 to assess type I error. We further assigned β = 0.3, γ = 0.2 for scenario 2 and β = 0.3, γ = 0.6 for both scenario 3 and scenario 4 to assess the power. Again, the effecting nodes and edges in these three nonlinear scenarios were also randomly selected.

For each scenario, 1000 replicates were used to evaluate the performance of type I error and power under different sample sizes (300, 400, 500, 600, 1000). We further designed four other scenarios under the same settings as above, except that the changing node and edges are fixed rather than randomly selected for each replicate.

### Applications

We first applied PMINR to analyze the gene expression data on lung cancer, available from Gene Expression Omnibus (GEO) with accession number GDS2771. Among the 187 smokers 97 were diagnosed with lung cancer and 90 were controls. The gene regulatory network of lung cancer from KEGG database involves 20 genes and 23 edges. Many probe sets corresponding to the same gene symbol were averaged to obtain gene-level expression measurement. We aimed to determine whether the whole pathway or gene or between-gene correlation can contribute to lung cancer development in smokers.

We then applied PMINR to the gene methylation data from the ROSMAP study as divided into two parts, ROS (The Religious Orders Study) and The Memory and Aging Project (MAP). The ROS is a longitudinal clinical-pathologic cohort study of aging and Alzheimer disease (AD; Bennett et al., 2012a). Memory and Aging Project is a longitudinal, epidemiologic clinical-pathologic cohort study of common chronic conditions of aging with an emphasis on decline in cognitive and motor function and risk of AD (Bennett et al., 2012b). Both cohorts were run from Rush University. Alzheimer disease status was determined by a computer algorithm based on cognitive test performance with a series of discrete clinical judgments made in series by a neuropsychologist and a clinician. Methylation data was generated on prefrontal cortex samples collected from deceased subjects from the ROS and MAP studies using the Illumina HumanMethylation450 BeadChip. These data have undergone a quality control analysis and have been adjusted for age, sex, and experimental batch effect. An extensive description of the QC and adjustment process are provided (De Jager et al., 2014). We mapped the DNA methylation data on the AD pathway (hsa05010) from KEGG, including a total of 22 genes and 24 edges. The methylation level for one specific gene was calculated by averaging the corresponding *beta* value along this gene, including the gene body and upstream regions. Thus, for each individual, we had 22 gene methylation variables, the 267 cases are subjects with diagnosed AD and no other causes of cognitive impairment, and the 235 controls are those categorized as no cognitive impairment. We aimed to determine whether the whole pathway or gene methylation or between-gene methylation correlation can contribute to AD development.

## Results

### Simulation

**FIGURE 2:**
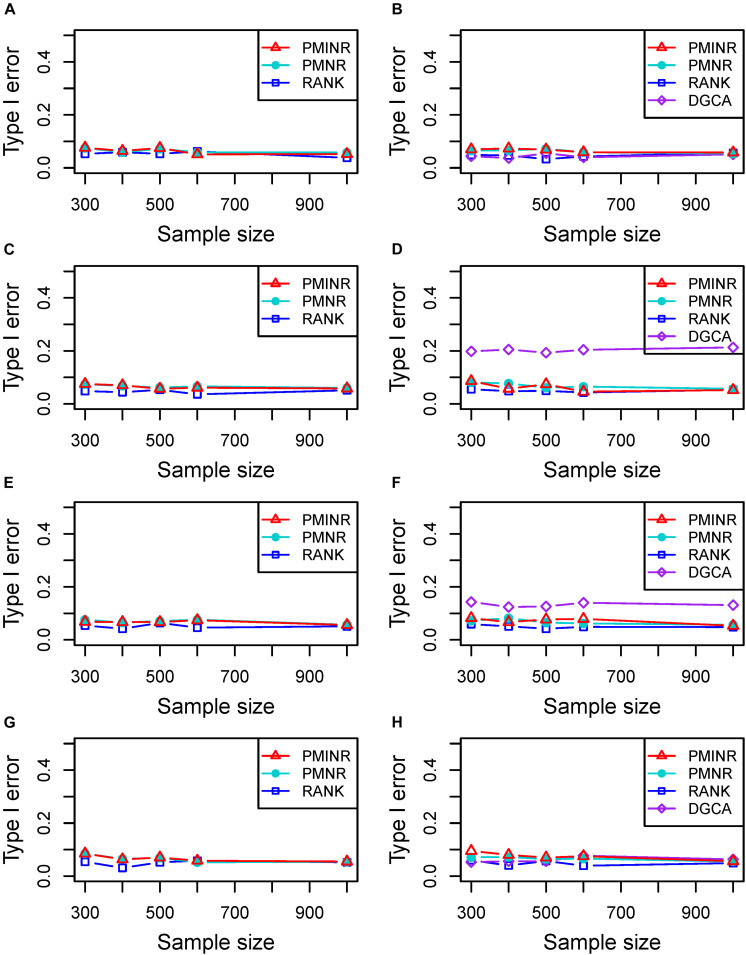
Type I error of PMINR, PMNR, RANK and DGCA. **(A)** the result for detecting node under scenario 1, **(B)** detecting edge under scenario 1, **(C)** detecting node under scenario 2, **(D)** detecting edge under scenario 2, **(E)** detecting node under scenario 3, **(F)** detecting edge under scenario 3, **(G)** detecting node under scenario 4, **(H)** detecting edge under scenario 4.

Shown in Figure 2 are the estimated type I error rates of the four methods. For detecting the effecting node, the type I error rates of all methods are close to given nominal level (α = 0.05) when the sample size is relatively large, regardless of the correlation pattern being linear (Figure 2A), quadratic (Figure 2C), sine (Figure 2E) or the recombination of quadratic and sine (Figure 2G). While all the methods are a little inflated for the small sample size (e.g., 300). In addition, similar trends of type I error rates can be found for detecting the effecting edge. Note that when the nonlinear pattern is quadratic or sine, DGCA has much higher type I error rates than any other method in detecting the effecting edge (Figures 2D,F), which is because DGCA can only capture the linear relationship and may be unable to reflect the nonlinear correlation. Similar trends of type I error rates can also be found when the effecting node and edge are fixed (Supplementary Figure 1).

**FIGURE 3:**
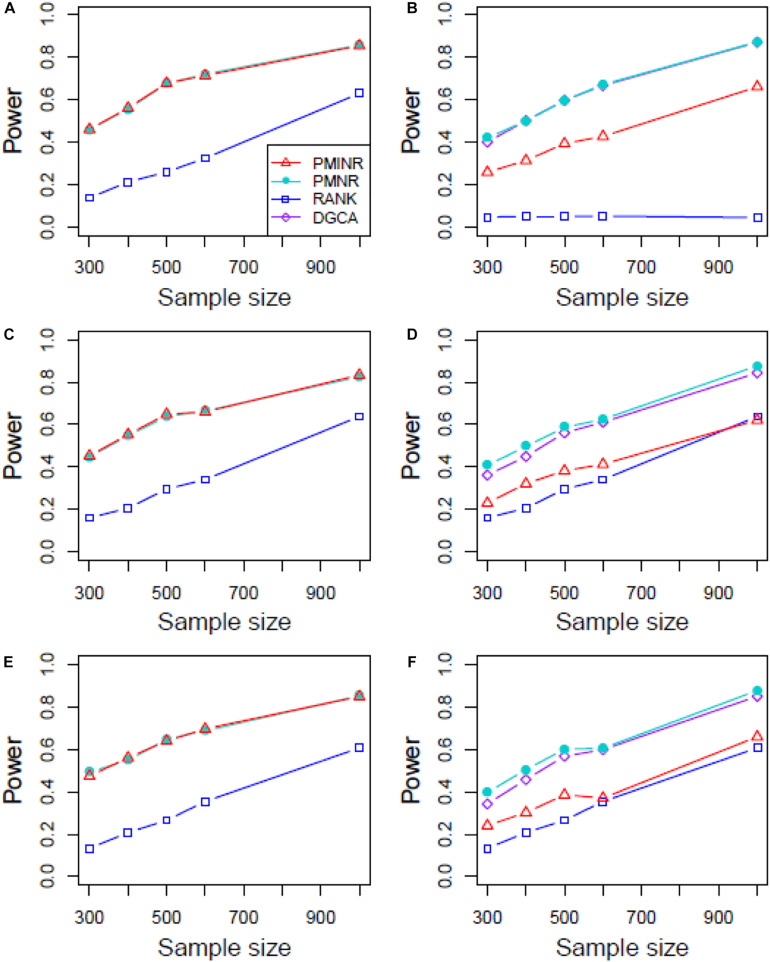
The statistical power of PMINR, PMNR, RANK and DGCA under scenario 1. **(A)** Only node changes. **(B)** Only edge changes. Both node and edge change, with effecting node hanging on the edge, **(C)** the result for effecting node, **(D)** the result for effecting edge. Both node and edge change with node not hanging on the edge, **(E)** the result of effecting node, **(F)** the result of effecting edge. Note that the power of DGCA to test the effecting node is not presented due to DGCA conceptually only capture the effecting edge.

Shown in Figure 3 are the power of the four methods under scenario 1 when all the correlation patterns are linear. The power of all methods increases with sample size. In detection of the effecting node, PMINR and PMNR have the highest power than the other methods regardless of only node effecting (Figure 3A) or both node and edge effecting (Figures 3C,E). The RANK method has relatively lower power possibly due partly to RANK test as being essentially nonparametric and only able to give the overall *p* value for the global network without little ability to identify the specific effecting node or edge. To detect the effecting edge, PMINR is expected to have lower power than PMNR and DGCA in this case, both of which are the gold standard and have comparable power under various situations (Figures 3B,D,F).

**FIGURE 4:**
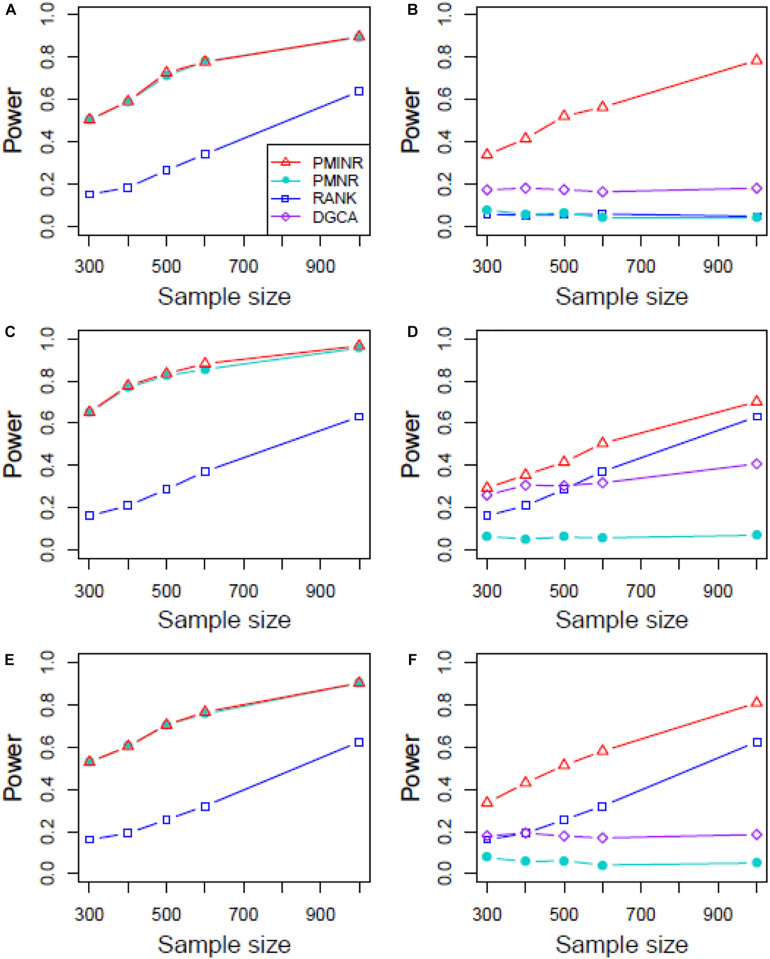
The statistical power of PMINR, PMNR, RANK and DGCA under scenario 2. **(A)** Only node changes. **(B)** Only edge changes. Both node and edge change, with effecting node hanging on the edge, **(C)** the result for effecting node, **(D)** the result for effecting edge. Both node and edge change with node not hanging on the edge, **(E)** the result of effecting node, **(F)** the result of effecting edge. Note that the power of DGCA to test the effecting node is not presented due to DGCA conceptually only capture the effecting edge.

**FIGURE 5:**
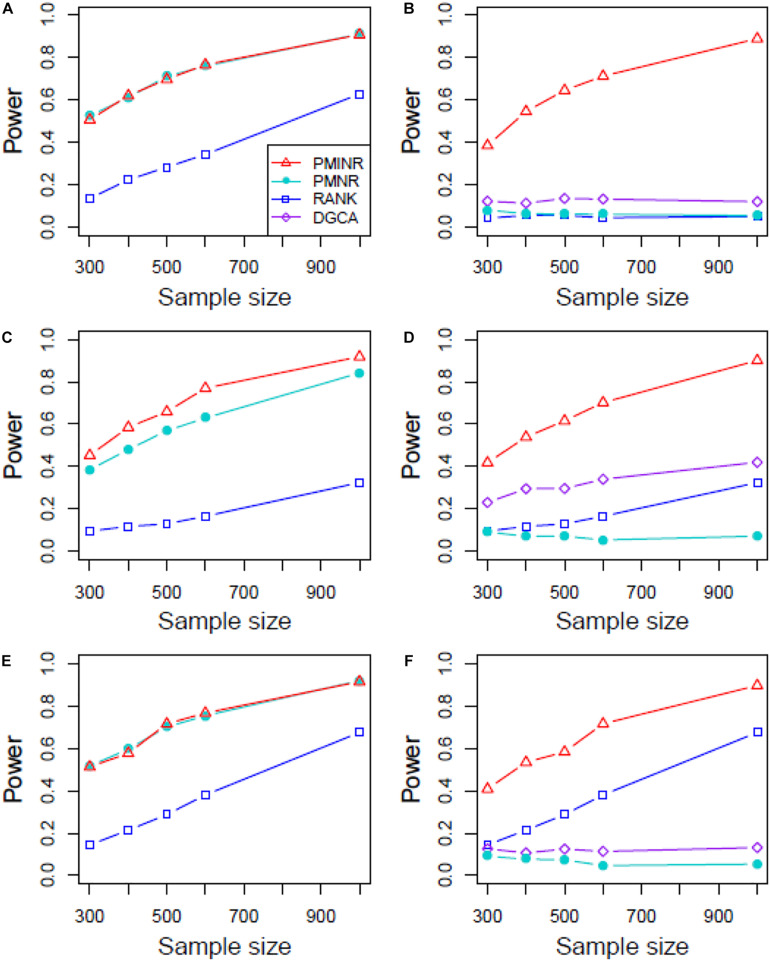
The statistical power of PMINR, PMNR, RANK and DGCA under scenario 3. **(A)** Only node changes. **(B)** Only edge changes. Both node and edge change, with effecting node hanging on the edge, **(C)** the result for effecting node, **(D)** the result for effecting edge. Both node and edge change with node not hanging on the edge, **(E)** the result of effecting node, **(F)** the result of effecting edge. Note that the power of DGCA to test the effecting node is not presented due to DGCA conceptually only capture the effecting edge.

**FIGURE 6:**
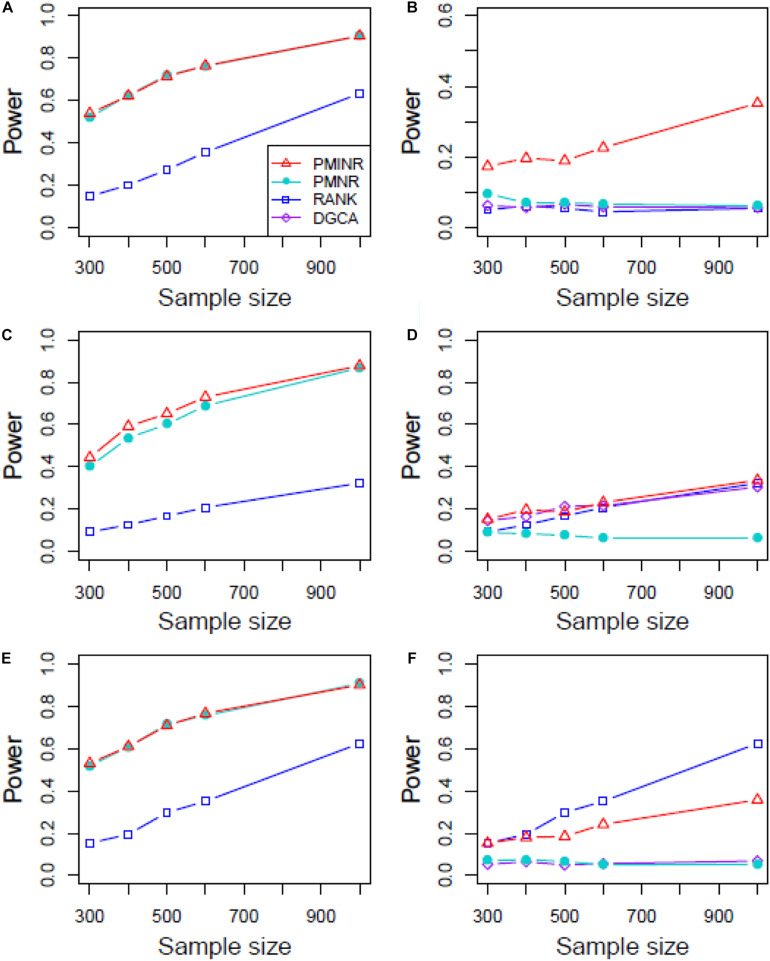
The statistical power of PMINR, PMNR, RANK and DGCA under scenario 4. **(A)** Only node changes. **(B)** Only edge changes. Both node and edge change, with effecting node hanging on the edge, **(C)** the result for effecting node, **(D)** the result for effecting edge. Both node and edge change with node not hanging on the edge, **(E)** the result of effecting node, **(F)** the result of effecting edge. Note that the power of DGCA to test the effecting node is not presented due to DGCA conceptually only capture the effecting edge.

Figures 4–6 show the power of all methods when the correlation pattern is nonlinear (scenario 2, 3, and 4 in the above simulation settings). In identification of the effecting node, PMINR has the highest power regardless of whether the nonlinear pattern is quadratic (Figures 4A,C,E), sine (Figures 5A,C,E) or the recombination of quadratic and sine (Figures 6A,C,E). Note that PMNR have the comparable power in detecting the effecting node under almost all situations, except when both node and edge change with the effecting node hanging on the edge (Figures 5C, 6C). This is partly because PMNR conceptually only captures the linear relationship, and the nonlinear correlation on the edge can affect the power to detect the effecting node hanging on itself. In detection of the effecting edge, PMINR has the highest power under almost all the situations, except when both node and edge change with the effecting node not hanging on the edge under scenario 4 (Figure 6F). While in such cases, the power of the RANK method is higher than that of PMINR. This may be partly due to PMI having little ability to capture and reflect the nonlinear relationship of the recombination of quadratic and sin. In addition, both PMNR and DGCA substantially lose power since they are unable to capture the nonlinear relationship. In addition, similar phenomenon can be found when the effecting node and edge are set to be fixed rather than randomly selected (Supplementary Figures 2–5). Overall, the performance of PMINR is at least comparable, and often superior, to that of existing methods.

### Applications

Shown in Table 1 are the results of lung cancer data based on the non-small cell lung cancer (NSCLC) pathway. Consistent with the simulation results, PMINR have successfully detected more genes and edges than the other methods at a significance level of 0.05. Both PMINR and PMNR have identified two common genes (*BAD* and *JAK3*). In addition, one significant edge (*CASP9*-*AKT2*) has been also identified by PMINR and DGCA. Again, the RANK method can only present the overall *p* value for the global pathway.

**TABLE 1:** Lung cancer network regression of various methods with *p* values in parenthesis.

Method	Edge	Node
PMINR	*ERBB2-TGFA (0.0069); PIK3CD-EML4 (0.019); PIK3CD-AKT2 (0.034); ERBB2-PIK3CD (0.037); RAF1-MAP2K1(0.047); AKT2-CASP9 (0.049)*	*BAD (0.0003); JAK3 (0.019); AKT2 (0.031); EGF (0.035)*
PMNR		*BAD (0.0057); JAK3 (0.015)*
DGCA	*JAK3*-*STAT3 (0.0087); AKT2*-*CASP9 (0.016)*	
RANK	global network (0.022)	global network (0.022)

Shown in Table 2 are the results of the ROSMAP data based on the AD pathway. At the significance level of 0.05, both PMINR and PMNR have identified the same gene methylation nodes (*CDK5, MAPK1, GRIN2A*), which indicated that, consistent with the simulation results, PMINR and PMNR have the comparable power to detect the node effects. The results for detecting the edge effect are quite different from other methods. However, if treating the DGCA method as the gold standard capturing the linear relationship, we found that those edges with more linear relationships can be almost significantly detected by PMINR, but not vice versa. For example, the p values for *FAS-FADD* and *CAPN1-CDK5R1* are 0.018 and 0.027, respectively for DGCA, and 0.059 and 0.062 for PMINR. The p values for *CALM1-PPP3CA* and *CASP12-CASP3* are 0.010 and 0.045, respectively for PMINR, but 0.485 and 0.854, respectively for DGCA.

**TABLE 2:** AD network regression of various methods with p values in parenthesis.

Method	Edge	Node
PMINR	*CALM1*-*PPP3CA (0.0010); CASP12-CASP3 (0.045)*	*CDK5(0.011); MAPK1(0.030); GRIN2A (0.011)*
PMNR	*CASP8*-*CASP3(0.013); MAPT-CDK5 (0.024)*	*CDK5(0.0069); MAPK1(0.034); GRIN2A (0.036)*
DGCA	*FAS-FADD (0.018); CASP8*-*CASP3(0.0058); CAPN1*-*CDK5R1(0.027)*	
RANK	global network (0.012)	global network (0.012)

It should be noted that under Bonferroni correction, only *BAD* is significant in lung cancer data, while no significantly effecting nodes or edges can be found in ROSMAP data. It may not be straightforward to correct for multiple comparison given the high level of correlation between tests, and the commonly used Bonferroni correction may be too stringent.

## Discussion

In recognition of the importance of biological networks as in complex diseases (Barabási et al., 2011) and their use in identification of high-risk genes and pathways therefore drug development, we have developed PMINR to account for group difference of biological networks due not only the effect of nodes but the effect of edges. We first introduced PMI to measure the connection between two nodes, then proposed PMINR model to differentiate patterns of network changes (node change or edge change) responsible for a disease outcome. One strong argument is that besides Pearson correlation many non-parametric and robust correlation measures such as distance correlation, mutual information and maximal information coefficient may also be chosen to depict the network inter-node connection. Often for a given sample, one can only calculate one unique correlation value using these measures. Moreover, in regression framework, each sampled individual should have its own correlation. PMI can be used directly in regression, and more attractively capture both linear relationships and nonlinear correlation. Extensive simulations showed that PMINR has better performance than other available methods.

Findings from the NSCLC dataset are consistent with earlier reports. Increasing expression of *BAD* enhances apoptosis and has a negative influence on cell proliferation and tumor growth in NSCLC (Jiang et al., 2013). The *JAK3* gene is confirmed to be associated with lung cancer (Yoo et al., 2007). Zyuz’kov et al. (2016) found a pronounced inhibition of hematogenous spread of the pathologic process into lungs, and blockade of *JAK3* significantly elevated maturation index of the tumor tissue. Moreover, *Akt2* and *CASP9* play an important role in lung cancer progression (Park et al., 2006; Wang et al., 2006; Lou et al., 2007; Attoub et al., 2015). In fact, increasing evidence points to the functional importance of alternative splice variations in cancer pathophysiology, and Shultz et al. (2010) found that oncogenic factors activating the PI3Kinase/AKT pathway can regulate alternative splicing of *CASP9* via a coordinated mechanism involving the phosphorylation of *SRp30a*. It implies that there may be an interaction between *CASP9* and *AKT2* in the progression of lung cancer.

The systemic failure of calmodulin degradation, and thus of Ca(2+)/ calmodulin dependent signaling pathways, may be important in the etiopathogenesis of AD. Both *CALM1* and *PPP3CA* play essential roles in the transduction of intracellular Ca(2+)-mediated signals, in that *CALM1* encodes calcium binding protein which is a subunits of phosphorylase kinase and can bind *PPP3CA* regulatory domain and causes a conformational change in removing *PPP3CA* autoinhibitory domain from its catalytic site, i.e., activating *PPP3CA* (Dunlap et al., 2013). In addition, Activated *CASP3* may be a factor in functional decline and may have an important role in neuronal cell death and correlation with Alzheimer pathology (Su et al., 2001; Gastard et al., 2003). *CDK5* has multiple roles in neuron development, neuronal survival, phosphorylation of cytoskeletal proteins and synaptic plasticity. Indeed, *CDK5* is reported to be intimately associated with the process of the pathogenesis of AD (Shukla et al., 2012; Liu et al., 2016). *MAPK1* encodes a member of the MAP kinase family. *MAPK1* is confirmed to be associated with the formation of hyperphosphorylated tau protein early in the development of AD (Gerschütz et al., 2014).

The apparent limitation in assuming known biological network structure can actually be useful for learning network structure which determines every possible edge with the highest degree of data matching, and a joint probability distribution of network nodes can reflect more than one network structure. Often, most biologists can roughly describe more or less the specific network for the corresponding biological process, and facilitated by multiple databases (such as KEGG) to establish the network structure. The inference of PMINR directly plugs the estimate of inter-node correlation into the regression model and fails to account for the uncertainty during inter-node correlation estimate. It should be noted that such inference procedure may lead to the biased estimate and power loss, especially in smaller sample size. The p values at present study are without accounting for the multiple testing. Often, the node test and the edge test are often highly correlated, and it is not straightforward to correct the p value or control the false discovery rate. However, not taking the multiple testing into account may make the interpretation of the results unclear, given that the truth is often unknown in practice. It is desirable to develop methods that can calculate the effective number of independent tests, to further address the multiple testing issue. In addition, caution should be used against the interpretation of estimated individual node and edge effects, given the potential for statistical mediation of effects within the network.

In conclusion, PMI captures the general inter-node correlation pattern in biological networks, and PMINR is powerful and efficient for biological network analysis.

## Data Availability Statement

Publicly available datasets were analyzed in this study. The datasets analyzed for this study can be found in the GEO with accession number GDS2771 and ROSMAP (https://www.synapse.org/#!Synapse:syn3219045).

## Author Contributions

ZY conceived the study. JJ and WL contributed to the data analysis. YZ, ML, FX, and JZ contributed to the data interpretation. ZY, WL, and JJ wrote the manuscript with help from JZ. All authors contributed to the article and approved the submitted version.

## Conflict of Interest

The authors declare that the research was conducted in the absence of any commercial or financial relationships that could be construed as a potential conflict of interest.
