# Supplementary material for: PMINR: Pointwise Mutual Information-Based Network Regression – With Application to Studies of Lung Cancer and Alzheimer’s Disease
Source: Front Genet. 2020 Oct 15;11:556259. doi: 10.3389/fgene.2020.556259 (PMC7594515; doi:10.3389/fgene.2020.556259)
Supplement: Supplementary file 1 [file Data_Sheet_1.pdf]

Supplementary Material

PMINR: pointwise mutual information-based network regression – with application to studies of lung cancer and Alzheimer's disease

Weiqiang Lin[1+], Jiadong Ji[2+], Yuchen Zhu[1], Mingzhuo Li[1], Jinghua Zhao[3], Fuzhong Xue[1], Zhongshang Yuan[1*]

[1]Department of Biostatistics, School of Public Health, Cheeloo College of Medicine, Shandong University, Jinan, Shandong, 250012, China.

[2]Department of Data Science, School of Statistics, Shandong University of Finance and Economics, Jinan 250014, China.

[3]Cardiovasucular Epidemiology Unit, Depart of Public Health and Primary Care, University of Cambridge, Cambridge CB1 8RN, UK.

*To whom correspondence should be addressed.

[+]These authors contributed equally to this work.

*** Correspondence:**
Zhongshang Yuan
yuanzhongshang@sdu.edu.cn

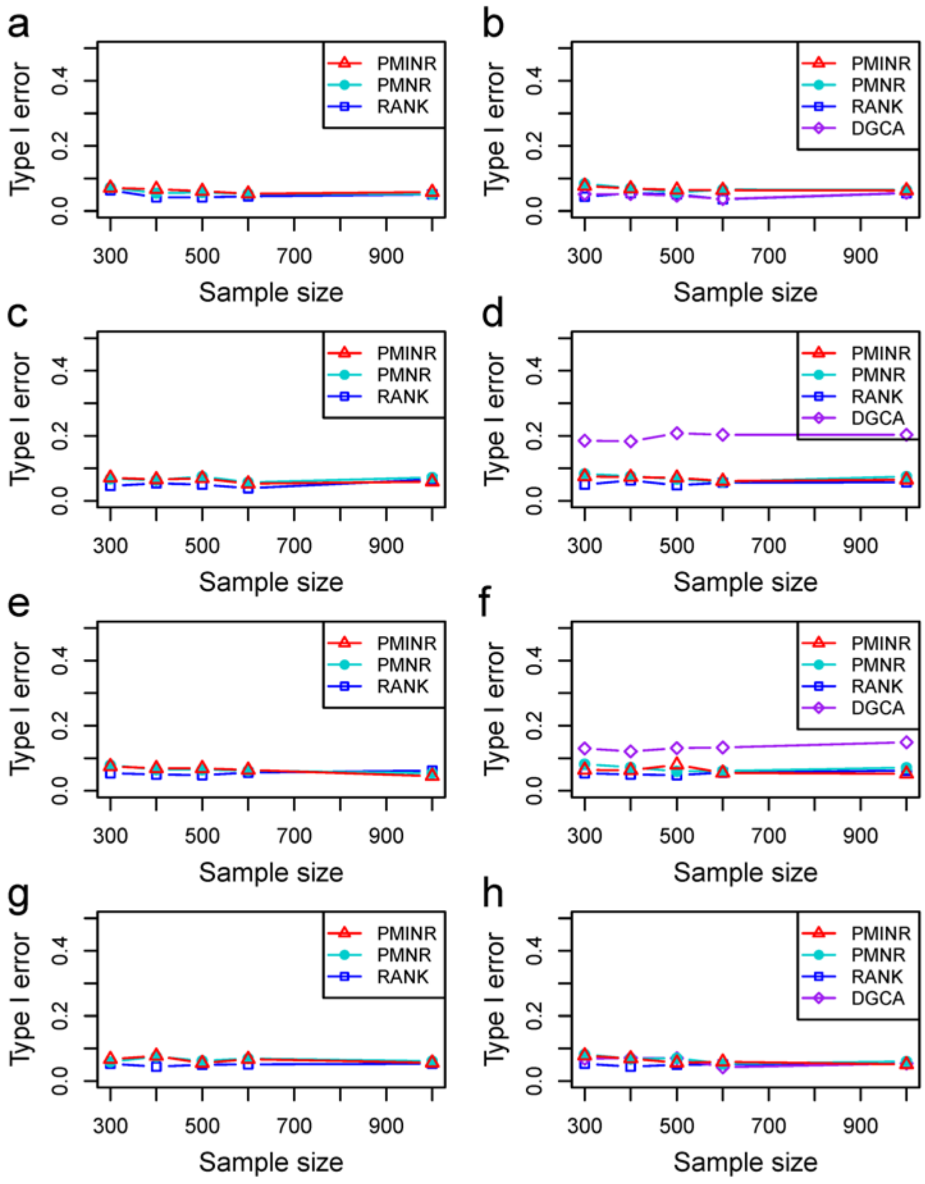

Figure S1. Type I error of PMINR, PMNR, RANK and DGCA when the changing node and edge are fixed. **(a)** the result for detecting node under scenario 1, **(b)** detecting edge under scenario 1, **(c)** detecting node under scenario 2, **(d)** detecting edge under scenario 2, **(e)** detecting node under scenario 3, **(f)** detecting edge under scenario 3, **(g)** detecting node under scenario 4, **(h)** detecting edge under scenario 4.

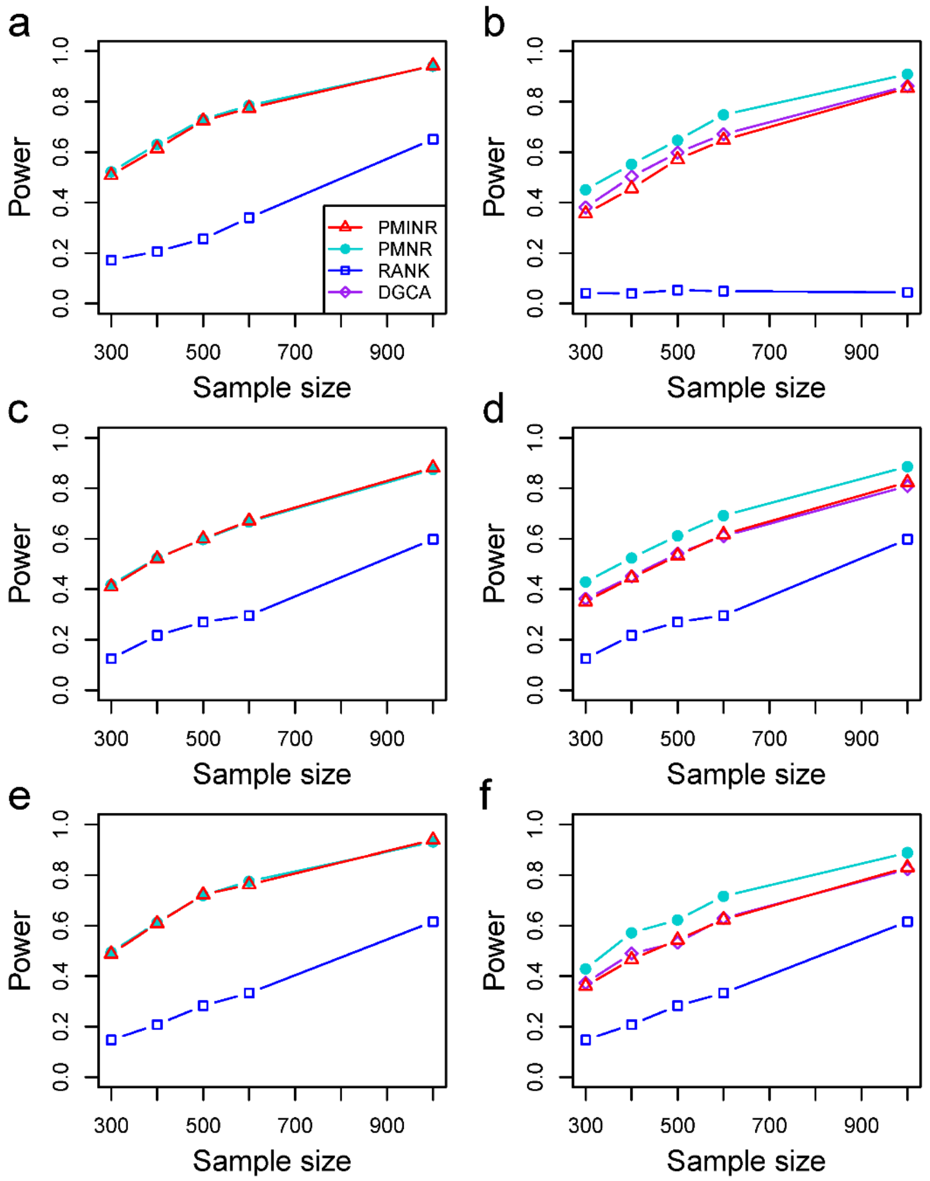

Figure S2. The statistical power of PMINR, PMNR, RANK and DGCA under scenario 1 when the changing node and edge are fixed. **(a)** only node changes, **(b)** only edge changes, both node and edge change, with effecting node hanging on the edge (**c**: the result for effecting node, **d**: the result for effecting edge), both node and edge change with node not hanging on the edge (**e**: the result of effecting node, **f**: the result of effecting edge). Note that the power of DGCA to test the effecting node is not presented due to DGCA conceptually only capture the effecting edge.

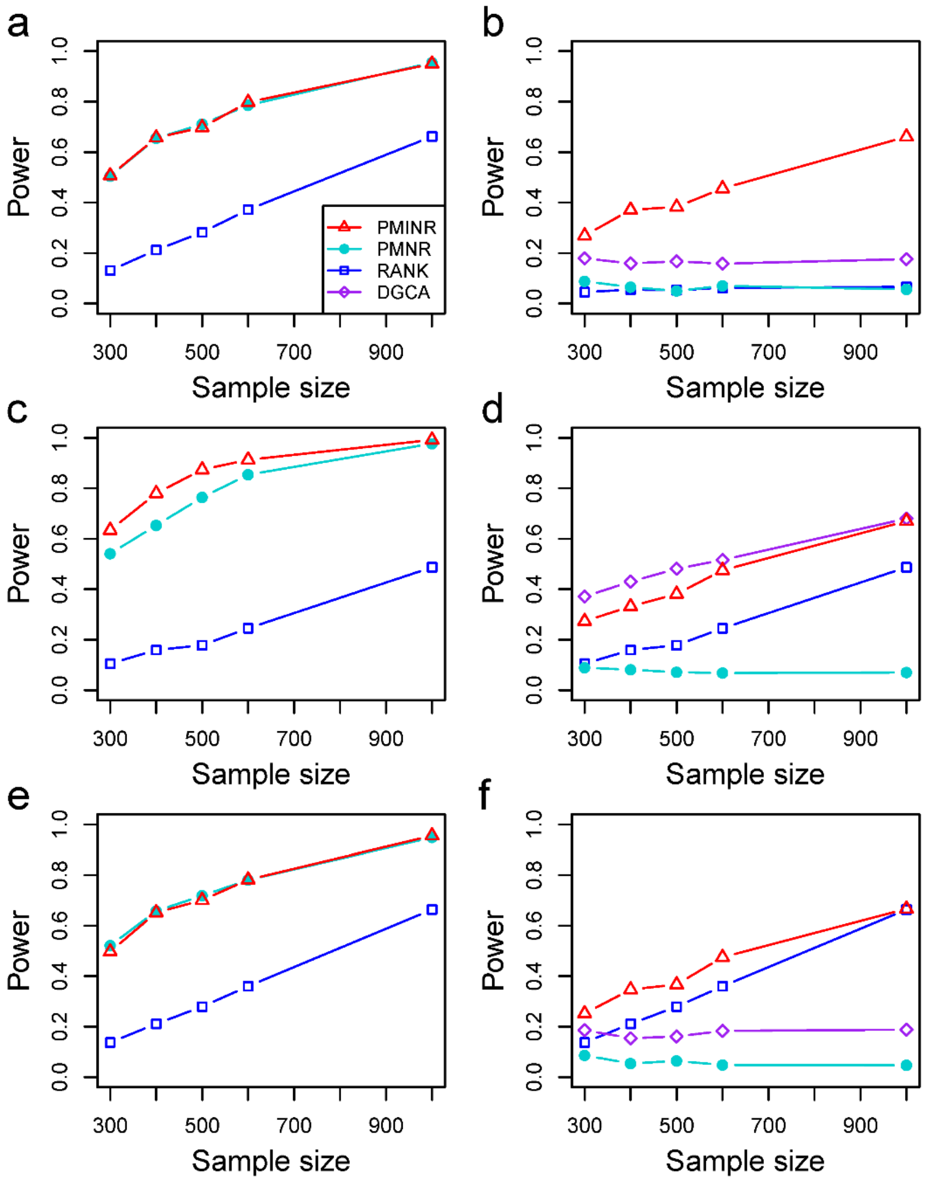

Figure S3. The statistical power of PMINR, PMNR, RANK and DGCA under scenario 2 when the changing node and edge are fixed. **(a)** only node changes, **(b)** only edge changes, both node and edge change, with effecting node hanging on the edge (**c**: the result for effecting node, **d**: the result for effecting edge), both node and edge change with node not hanging on the edge (**e**: the result of effecting node, **f**: the result of effecting edge). Note that the power of DGCA to test the effecting node is not presented due to DGCA conceptually only capture the effecting edge.

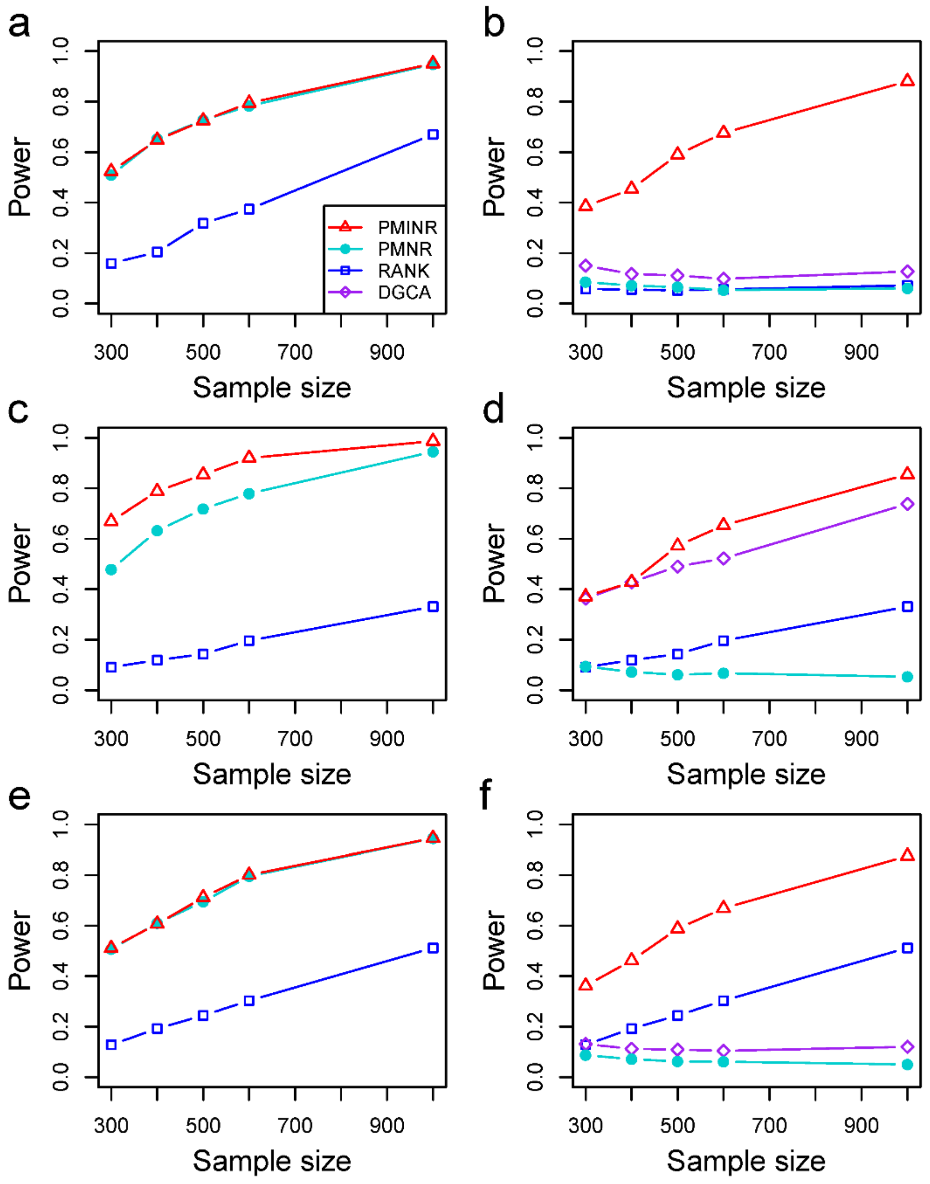

Figure S4. The statistical power of PMINR, PMNR, RANK and DGCA under scenario 3 when the changing node and edge are fixed. **(a)** only node changes, **(b)** only edge changes, both node and edge change, with effecting node hanging on the edge (**c**: the result for effecting node, **d**: the result for effecting edge), both node and edge change with node not hanging on the edge (**e**: the result of effecting node, **f**: the result of effecting edge). Note that the power of DGCA to test the effecting node is not presented due to DGCA conceptually only capture the effecting edge.

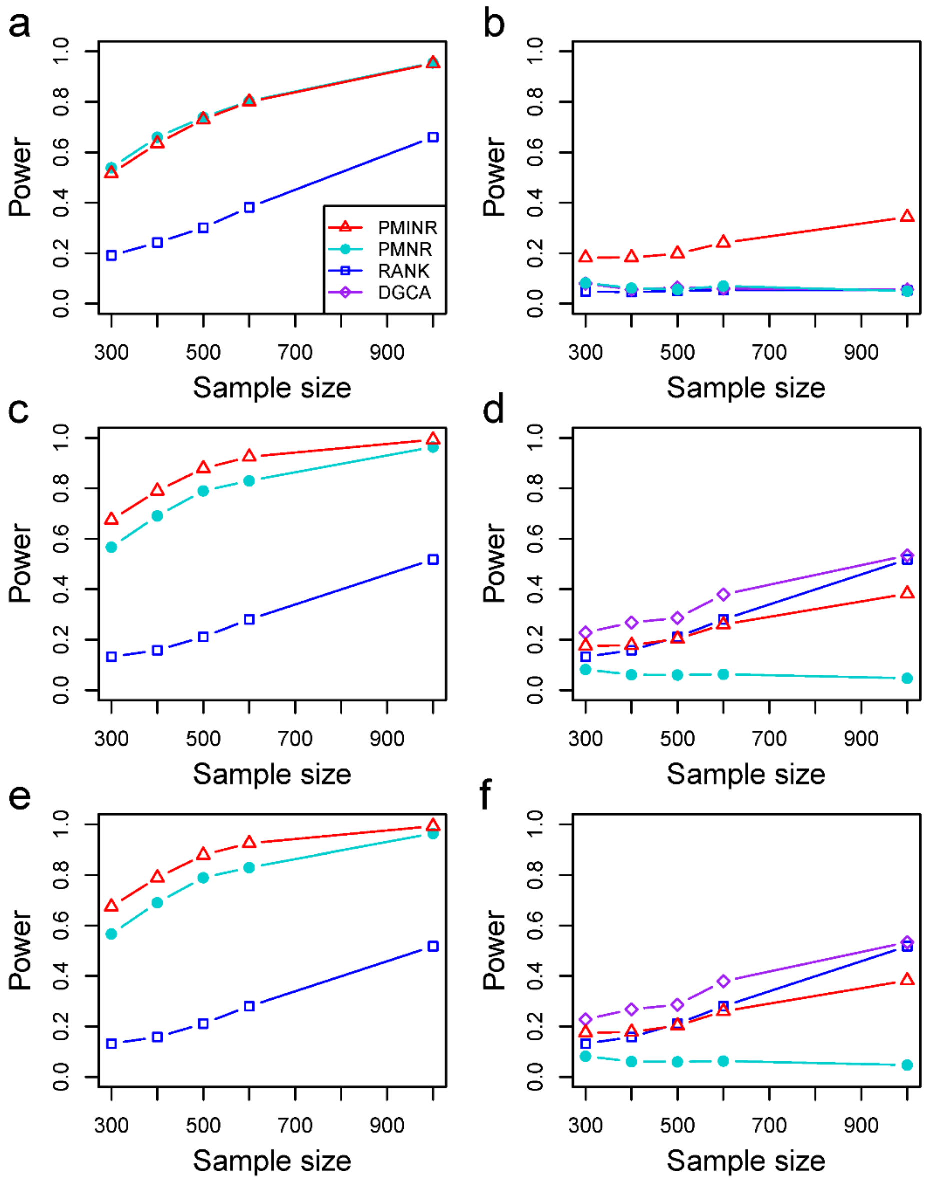

Figure S5. The statistical power of PMINR, PMNR, RANK and DGCA under scenario 4 when the changing node and edge are fixed. **(a)** only node changes, **(b)** only edge changes, both node and edge change, with effecting node hanging on the edge (**c**: the result for effecting node, **d**: the result for effecting edge), both node and edge change with node not hanging on the edge (**e**: the result of effecting node, **f**: the result of effecting edge). Note that the power of DGCA to test the effecting node is not presented due to DGCA conceptually only capture the effecting edge.
